# Supplementary material for: Efficacy of the FIBROWALK Multicomponent Program Moved to a Virtual Setting for Patients with Fibromyalgia during the COVID-19 Pandemic: A Proof-of-Concept RCT Performed Alongside the State of Alarm in Spain
Source: Int J Environ Res Public Health. 2021 Sep 30;18(19):10300. doi: 10.3390/ijerph181910300 (PMC8508552; doi:10.3390/ijerph181910300)
Supplement: Supplementary file 1 [file ijerph-18-10300-s001.zip › ijerph-1346320-supplementary.pdf]

Table S1. Descriptive statistics and between-group analyses for primary and secondary outcomes in completers approach (without imputation of missing data).

	**FIBROWALK + TAU (*n*=75)**		**TAU (*n*=76)**		***F***	***p***	***d***
	**Baseline**	**Post**	**Baseline**	**Post**			
**Primary Outcome, M ± SD**							
FIQR	68.97 ± 15.94	61.68 ± 19.53	72.72 ± 16.08	72.98 ± 17.86	9.272	**0.003**	0.468
**Secondary Outcomes, M ± SD**							
TSK	28.07 ± 7.71	22.78 ± 7.71	29.11 ± 8.13	29.08 ± 7.45	20.938	**<0.001**	0.656
HADS Anxiety	12.65 ± 4.90	11.50 ± 5.01	12.86 ± 4.30	13.21 ± 4.67	6.025	**0.016**	0.328
HADS Depression	11.48 ± 5.18	9.39 ± 5.53	12.18 ± 4.52	12.32 ± 4.73	11.498	**0.001**	0.463
SF-PF	29.02 ± 15.97	47.61 ± 20.73	30.99 ± 18.28	33.94 ± 18.55	24.782	**<0.001**	0.892

Note: Statistically significant effects are **shown** in bold ($p \leq 0.05$). When the Benjamini–Hochberg correction was applied to correct for multiple comparisons, all significant effects remained significant. FIQR: Revised Fibromyalgia Impact Questionnaire; HADS: Hospital Anxiety and Depression Scale; ISPS: Illness Self-Perceived Start; SF-PF: Physical Functioning component of the 36-Item Short Form Survey; TSK: Tampa Scale for Kinesiophobia.
